# The Role of ATR Inhibitors in Ovarian Cancer: Investigating Predictive Biomarkers of Response

**DOI:** 10.3390/cells11152361

**Published:** 2022-08-01

**Authors:** Alice Bradbury, Frank T. Zenke, Nicola J. Curtin, Yvette Drew

**Affiliations:** 1 Molecular Therapeutics Program, Fox Chase Cancer Center, Philadelphia, PA 19111, USA; alice.bradbury@fccc.edu; 2 Translational Innovation Platform Oncology, Merck KGaA, Frankfurt Strasse 250, D-64293 Darmstadt, Germany; frank.zenke@merckgroup.com; 3 Newcastle University Center for Cancer, Translational and Clinical Research Institute, Faculty of Medical Sciences, Newcastle University, Newcastle upon Tyne NE2 4HH, UK; 4 BC Cancer Vancouver, Departments of Medicine and Obstetrics and Gynecology, Faculty of Medicine, University of British Columbia, Vancouver, BC V5Z 4E6, Canada; yvette.drew@bccancer.bc.ca

**Keywords:** ovarian cancer, ATR inhibitor, VE-821, monotherapy, replication stress

## Abstract

Ataxia telangiectasia and Rad-3 related kinase (ATR) signals DNA lesions and replication stress (RS) to the S and G2/M checkpoints and DNA repair pathways making it a promising target to exploit the dysregulated DNA damage response in cancer. ATR inhibitors (ATRi) are under clinical investigation as monotherapy and in combination with other anticancer agents. Molecular determinants of sensitivity to ATRi are common in ovarian cancer, suggesting the therapeutic potential of ATRi. We investigated the cytotoxicity of the ATRi, VE-821, in a panel of human ovarian cancer cell lines. High grade serous (HGS) cell lines were significantly more sensitive to VE-821 than non-HGS (*p* ≤ 0.0001) but previously identified determinants of sensitivity (*TP53*, *ATM* and *BRCA1*) were not predictive. Only low RAD51 (*p* = 0.041), TopBP1 (*p* = 0.026) and APOBEC3B (*p* = 0.015) protein expression were associated with increased VE-821 sensitivity. HGS cells had increased levels of RS (pRPA^Ser4/8^ and γH2AX nuclear immunofluorescence), and elevated RS predicted sensitivity to VE-821 independently of the cell line subtype. These data suggest that functional assessment of RS biomarkers may be a better predictive biomarker of ATRi response than any single aberrant gene in ovarian cancer and potentially other cancers.

## 1. Introduction

Ovarian cancer is the seventh most common cancer among women accounting for ~300,000 cancer cases globally in 2018 and is the leading cause of death from gynecological malignancy [1]. The majority of ovarian cancers are of epithelial origin and there are five histopathological subtypes: high-grade serous (HGS), low-grade serous (LGS), clear cell, endometrioid and mucinous [2]. The most common subtype, HGS, is characterized by high genomic instability and defects in DNA damage response (DDR) pathways, which drive the development and progression of the disease [3,4]. There is a high frequency (>50%) of homologous recombination repair (HRR) defects (HRD) in HGS ovarian cancer (HGSOC) [5].

The exploitation of DDR defects is a promising approach to cancer therapy, exemplified by the success of the synthetic lethality of poly-ADP ribose polymerase (PARP) inhibitors (PARPi) in cancers with HRD. The introduction of PARPi in the treatment of platinum sensitive HRD HGSOC has resulted in significant clinical benefits with prolongation of progression-free survival (PFS) [6,7,8,9]. However, patients whose cancers are ‘biomarker negative’ for HRD (around half of all HGSOC), gain minimal, short-lived responses or no response to maintenance single agent PARPi therapy. Even in patients who do respond, the development of PARPi resistance is common (Reviewed in [10]). Therefore, there is a need to identify new targets and new combinations of targeted therapies in this area.

Ataxia telangiectasia and Rad-3 related kinase (ATR) is a key DDR kinase, responsible for signaling single stranded DNA (ssDNA), principally due to replication stress (RS), to the S and G2/M checkpoints and DNA repair [11]. In cancer cells, G1 checkpoint control is commonly lost, which, coupled with frequent activation of oncogenes that drive replication, results in increased RS and hence an increased reliance on the S and G2/M checkpoints [12,13,14]. p53 plays a key role in G1 checkpoint control and *TP53* mutations (present in almost 100% of HGSOC [5]) could render cancers more sensitive to ATR inhibition than p53-proficient cells, however the data has been inconsistent [15,16,17,18]. Numerous other determinants of sensitivity to ATR inhibition have been identified such as; ATM [17,19,20,21,22], XRCC1 [23,24,25], TopBP1 [26], and CHK1 [27]. Of note to HGSOC, where >50% have HRD [5], the HRR pathway proteins RAD51 [25,28], and BRCA2 [25], have been implicated as determinants of sensitivity to ATR inhibition. Additionally, *CCNE1* (cyclin E) amplification, which drives S-phase entry is present in ~20% of HGSOC [5] has been identified as a determinant of sensitivity to ATR inhibition [15,29]. Defects in NHEJ have also been implicated in ovarian cancer [30], and linked to ATR inhibitor response [25]. Furthermore, ARID1A [31] and APOBEC3A/B [32,33] overexpression, both of which are linked to clear cell ovarian cancer [29,34,35], have been found to selectively sensitize to ATR inhibition. Therefore, ATRi monotherapy is likely to be a successful treatment strategy in ovarian cancer.

Putative biomarkers of response need to be sensitive and easy to measure for successful translation into clinical practice. However, unless a single gene/protein loss accounts for the response, clinical utility often fails due to the complexity of molecular characteristics of human cancer. This study aimed to investigate whether previously identified determinants of sensitivity to ATR inhibition in non-ovarian cancers would act as predictive biomarkers in in vitro models of ovarian cancer, and to investigate novel determinants of sensitivity. Sensitivity to the predecessor of the clinical ATRi berzosertib (M6620, VX-970, VE-822), VE-821 [36], was investigated in a panel of human ovarian cancer cell lines. Through functional assessment of RS, we sought to determine whether increased RS could be a predictive biomarker for sensitivity to VE-821 ATR inhibition.

## 2. Materials and Methods

### 2.1. Cell Lines and Culture Conditions

Human cancer cell lines were obtained from ATCC or ECACC, excluding the NUCOLL43 cell line which was derived de novo from a sample of malignant ascites that spontaneously immortalized [37], and the A2780, CP70 A2 and CP70 B1 cell lines; a kind gift from Dr Jane Plum and Prof. Robert Brown (Imperial College London, London, UK). Cells were cultured up to a maximum of 30 passages from purchase or authentication, in either RPMI (IGROV1, OAW42, ES-2, A2780, CP70 A2, CP70 B1 and NUCOLL43) or DMEM (COV318, CAOV3 and COV362) media supplemented with 10% FBS and incubated at 37 °C with 5% CO_2_. Cell lines were tested for mycoplasma every 2 months. Derivatives of the MMR deficient A2780-CP70 cell line, CP70 A2 and CP70 B1 were established by transfection with chromosome 3 containing; wild-type *hMLH1* (CP70 B1), or mutant *hMLH1* and were grown in media supplemented with 200 μg/mL Hygromycin B. The *BRCA1* mutant cell line, UWB1.289, and its *BRCA1* corrected derivative, UWB1.289 + Br1, were grown in 50% RPMI, 50% MEBM media supplemented with 20% FBS and 200 μg/mL G418S for UWB1.289 + Br1 cells.

Classification of the panel of cell lines into “HGS” or “non-HGS” was made according to the characterization by Domcke et al. [38], which assessed HGS features including: *TP53* mutation, high CNA frequency, and a low frequency of mutations overall (occurring in only *TP53*, *BRCA1*, *BRCA2* or *RB1* most commonly). As well as features of non-HGS subtypes (i.e., low-grade serous, mucinous, endometrioid or clear cell) including wild-type *TP53*, closer to normal gene copy numbers, mutations in *PIK3CA*, *PTEN*, *KRAS*, *BRAF*, *CTNNB1* and *ARID1A*, and CNAs in *ERBB2.* Cell lines not included in the assessment by Domcke et al. [38], UWB1.289 and NUCOLL43, were classified using the same parameters.

### 2.2. Cytotoxicity Determination by Colony Formation Assay

Low densities of cells (50–4000 cells/well, depending on cell line plating efficiency) were seeded into 6-well plates and allowed to adhere for 24 h before being treated with 0–30 μM VE-821 for 48 h. The DMSO concentration was kept consistent at 0.5% in treated and untreated control cells. After 48 h, drug was removed and replaced with fresh media before cells were left to form colonies. After 9–21 days, cells were fixed in methanol: acetic acid (3:1 *V*/*V*) and stained in 0.4% crystal violet (*W/V*). Colonies were counted and percentage survival was calculated relative to the plating efficiency of treated vs. untreated DMSO controls. Interpolation of the survival curves was used to calculate the LC_50_, the concentration causing a 50% decrease in cell survival, of VE-821.

### 2.3. Measurement of Cellular Proteins by Western Blot

Lysates were collected from untreated cells at 70% confluence with RIPA buffer containing 1% protease inhibitor cocktail (Thermo Fisher, Waltham, MA, USA), following manufacturer instructions. Protein content was estimated by Pierce BCA assay (Thermo Fisher) following manufacturers guidelines, then samples were diluted to equal concentrations between 0.8–1 mg/mL in XT sample buffer and XT reducing agent (BioRad, Hercules, CA, USA), and boiled at 95 °C for 10 min. Lysates were separated by SDS-PAGE using 3–8% Tris-Acetate gels (BioRad), transferred to nitrocellulose Hybond^TM^ C membrane (Amersham, Buckinghamshire, UK), and blocked in 5% nonfat milk in TBS-T. Primary antibodies were incubated overnight at 4 °C in either 5% nonfat milk or 5% BSA in TBS-T. The following primary antibodies were used: ATM (Cell Signaling, Danvers, MA, USA, 2873; 1:500), ATR (Santa Cruz, Dallas, TX, USA, Sc515173; 1:200), ARID1A (Cell Signaling, 12354; 1:1000), CHK1 (Santa Cruz, Sc8408; 1:500), Cyclin E (Santa Cruz, Sc247; 1:500), DNA-PKcs (Santa Cruz, Sc390849; 1:500), Ku70 (Abcam, Cambridge, UK, Ab3114; 1:500), Ku80 (Abcam, Ab80592; 1:500), RAD51 (Santa Cruz, Sc8349; 1:1000), PARP1 (Biovision, Milpitas, CA, USA, 3001-100; 1:500), TopBP1 (Abcam, Ab2402; 1:1000) and XRCC1 (Santa Cruz, Sc56254; 1:500). HRP conjugated secondary antibodies (Agilent Dako, Santa Clara, CA, UKA, P0447 and P0448; 1:2000) were incubated in 5% nonfat milk for 1 h at room temperature. For detection, Clarity Western enhanced chemiluminescence substrate (BioRad) was added and bands were visualized using the G-box gel documentation system (Syngene, Cambridge, UK), quantified using ImageJ software Version 2.3.0/1.53q (NIH, Bethesda, MD, USA), and normalized to Ponceau S staining (Supplementary Figure S1). Ponceau-S was used in place of a housekeeping gene in the Western blots, as although the expression of housekeeping proteins is unlikely to vary between samples of the same cell line prepared at the same time, they are more likely to vary between cell lines [39,40].

### 2.4. Measurement of Replication Stress by Immunofluorescence Microscopy

Cells were seeded onto coverslips and allowed to establish exponential growth in drug-free medium for 24 h before the coverslips were washed once in ice cold PBS, permeabilized in 0.2% triton-X/PBS for one minute, and then fixed in 2% paraformaldehyde. After fixing cells were washed once in PBS before being washed 3 times in 0.2% BSA 0.3% triton-X/PBS. Cells were then blocked in 5% BSA + 1:25 goat serum/PBS for one hour at room temperature. After blocking, primary antibodies were incubated overnight at 4 °C in 0.2% BSA 0.3% triton-X/PBS. The following primary antibodies were used: pRPA^Ser4/8^ (Bethyl Laboratories, Montgomery, TX, USA, A300-245A; 1:4000) and γH2AX^Ser139^ (MilliporeSigma, Burlington, MA, USA #05-636; 1:1000). After primary antibody incubation Cells were washed once in 0.2% BSA 0.3% Triton-X/PBS before being washed three times in 0.2% BSA/PBS, then incubated with Alexa Fluor conjugated secondary antibodies (Thermo Fisher, A-11003 and A-11008; 1:1000) for 1 h at room temperature. Cells were washed once in 0.2% BSA/PBS before being incubated with DAPI diluted 1:1000 for 30 min at room temperature. After DAPI, cells were washed 3 times in 0.2% BSA/PBS then mounted onto slides with Prolong Glass Antifade mountant (Thermo Fisher) and imaged using a DM6 widefield microscope. Multiple images per cell line were captured in each independent experiment and analyzed using ImageJ software, Version 2.3.0/1.53q (NIH)).

## 3. Results

### 3.1. HGS Ovarian Cancer Cell Lines Are More Sensitive to Single Agent Ve-821 Than Non-Hgs Ovarian Cancer Cell Lines

**Figure 1:**
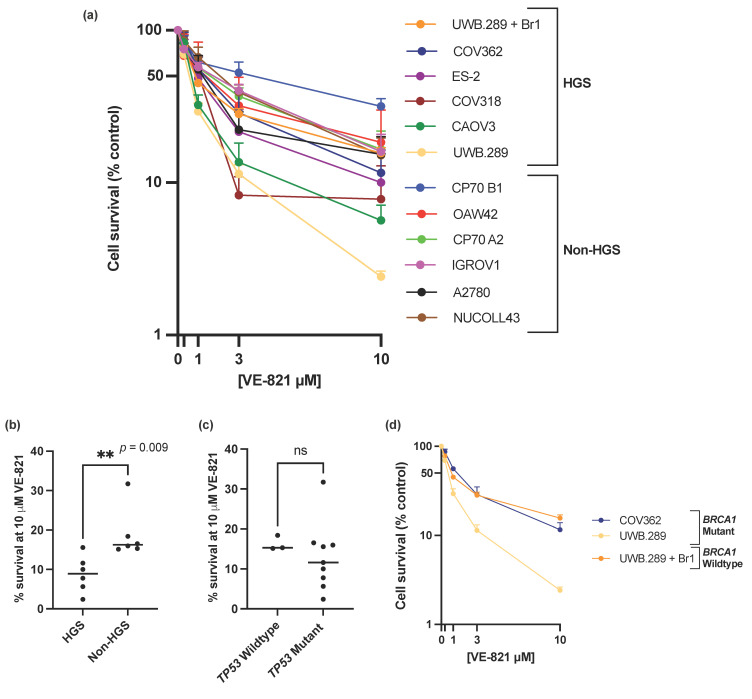
(**a**) Mean single agent cytotoxicity of VE-821 by colony survival assay. All data points are mean ± SEM (at least 3 independent experiments). (**b**) Median percentage cell survival at 10 μM VE-821 of HGS and non-HGS cell line is indicated with horizontal black line. ** *p* < 0.01 (Mann–Whitney test). (**c**) Median percentage cell survival at 10 μM VE-821 of *TP53* WT or mutant cell lines is indicated by horizontal black line. ns, *p* > 0.05 (Mann–Whitney test). *TP53* Wildtype cell lines; OAW42, NUCOLL43, A2780, *TP53* mutant cell lines; IGROV1, COV318, COV362, CAOV3, ES-2, UWB1.289, UWB1.289 + Br1, CP70 A2, CP70 B1. (**d**) Single agent cytotoxicity of VE-821 in *BRCA1* mutant cell lines COV362 and UWB1.289, and *BRCA1* corrected cell line UWB1.289 + Br1. All data points are mean ± SEM (at least 3 independent experiments).

ATRi VE-821 showed a broad spectrum of single agent activity across the panel of ovarian cancer cell lines (Figure 1a), with a 13-fold difference of percentage cell survival at 10 µM VE-821 between the least sensitive CP70 B1 (31.7% survival) and the most sensitive UWB1.289 (2.4% survival) (Figure 1a and Supplementary Table S1) and a 6-fold difference in the LC_50_ values between the CP70 B1 cell line (LC_50_ = 4.13 µM) and the UWB1.289 cell line (LC_50_ = 0.64 µM) (Supplementary Table S1). To demonstrate that the difference in sensitivity to VE-821 across the panel of cell lines was an ATRi class effect rather than VE-821 specific, sensitivity to the clinical ATRi candidate berzosertib was also investigated in the IRGOV1 and CAOV3 cells (Supplementary Table S2). The CAOV3 cells were 2–3x more sensitive to berzosertib than the IGROV1 cells, comparable to the difference in sensitivity to VE-821 between the two cell lines.

The data clearly shows that that HGS cell lines were significantly more sensitive to single-agent VE-821 than non-HGS cell lines (*p*-value = 0.009) (Figure 1b, Supplementary Figure S1a). However, single molecular aberrations in the cell line panel were not able to consistently predict sensitivity to VE-821. Mutations in *TP53*, previously proposed as a determinant of ATRi cytotoxicity [15,17], and almost ubiquitous in HGSOC [5], was not associated with increased VE-821 cytotoxicity (Figure 1c, Supplementary Figure S1b). The *BRCA1* mutant UWB1.289 cell line was significantly more sensitive to VE-821 than the *BRCA1* corrected counterpart UWB1.289 + Br1 (un-paired *t*-test of percentage cell survival at 10 µM VE-821, *p*-value ≤ 0.0001). However, VE-821 was not particularly cytotoxic in the homozygous *BRCA1* mutant COV362 cell line (Figure 1d) despite the cell line previously being shown to be HRD, and sensitive to PARPi (rucaparib) and carboplatin [41].

### 3.2. Reduced APOBEC3B, RAD51 and TopBP1 Protein Expression Was Associated with Increased Sensitivity to VE-821

**Figure 2:**
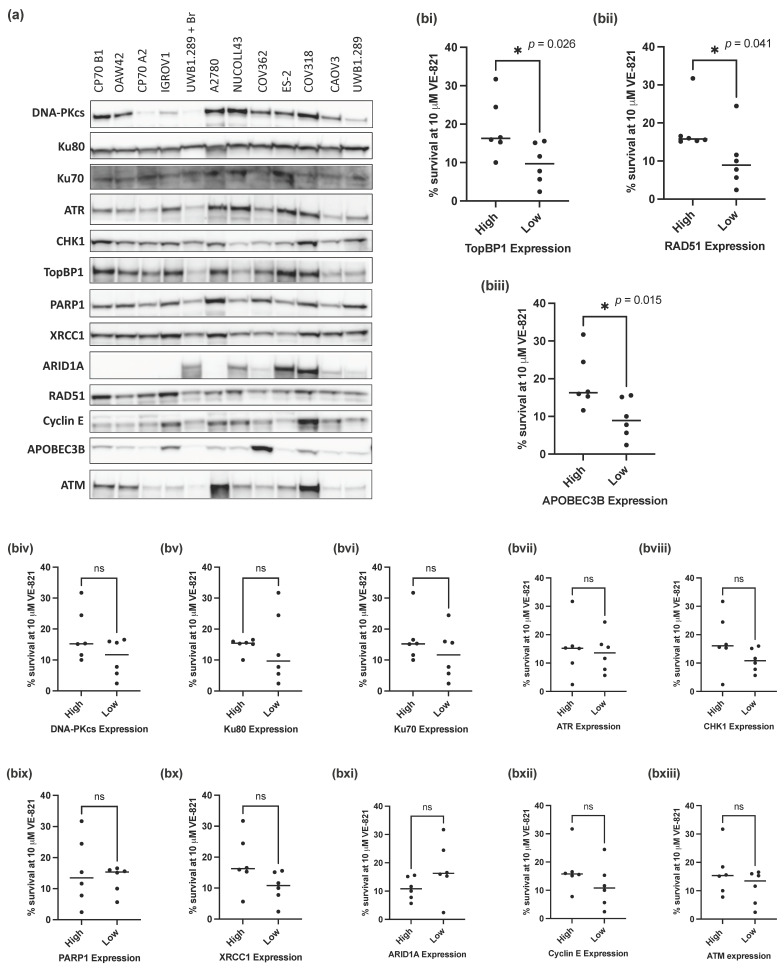
Measurement of previously identified determinants of sensitivity to ATRi. (**a**) Representative Western blot of 3 independent experiments. (**b**) Mean survival at 10 μM VE-821 of cell lines split into high (above the median) or low (below the median) normalized protein expression for each of the following proteins: (**i**) TopBP1, (**ii**) RAD51, (**iii**) APOEB3B, (**iv**) DNA-PKcs, (**v**) Ku80, (**vi**) Ku70, (**vii**) ATR, (**viii**) CHK1, (**ix**) PARP1, (**x**) XRCC1, (**xi**) ARID1A, (**xii**) Cyclin E, (**xiii**) ATM. Data points are individual cell lines. The horizontal black bar indicates median survival of each group of cell lines. * *p* < 0.05; ns, not significant (Mann–Whitney test).

Since mutations in *TP53* and *BRCA1* were not predictive of sensitivity to VE-821, we investigated whether expression of 13 key proteins (DNA-PKcs, Ku80, Ku70, ATR, CHK1, PARP1, XRCC1, ARID1A, RAD51, TopBP1, Cyclin E, APOBEC3B and ATM) (Figure 2a), previously identified as determinants of sensitivity to ATRi, would associate with the sensitivity of the panel of ovarian cancer cell lines to VE-821.

To assess the impact of different levels of protein expression on the sensitivity of the cell lines to VE-821, cell lines were classified as having high (above the cell line panel median) or low (below the median) protein expression for each of the 13 proteins analyzed. The percentage cell survival at 10 µM VE-821 of the cell lines with high or low protein expression was then assessed and compared for each individual protein. Of the 13 proteins analyzed, cells with low RAD51 (*p*-value = 0.041), TopBP1 (*p*-value = 0.026) and APOBEC3B (*p*-value = 0.015) protein expression (Figure 2(bi–biii)) had a significantly lower percentage cell survival at 10 µM VE-821 than those with high expression of either protein (Figure 2(biii–bxiii)). Contrary to expectation, there was no difference in survival associated with ATM levels (Figure 2(bxiii)).

Whilst statistical significance was not reached in the additional 10 proteins analyzed, cell lines with lower NHEJ protein expression (DNA-PKcs, Ku80 and Ku70) tended to have a lower percentage cell survival at 10 µM VE-821 (Figure 2(biv–bvi)).

ATRi and PARPi combinations have been shown to have synergistic cytotoxic activity in a variety of preclinical models [42,43,44] and are being evaluated in combination clinically [45]. We measured the expression of BER proteins PARP1 and XRCC1 in relation to VE-821 sensitivity (Figure 2(bix,x)); however, there was no difference in sensitivity to VE-821 between cells with high or low PARP1 expression. However, PARP enzymatic activity, which does not correlate well with protein expression [46], may be a better predictor of sensitivity to VE-821. Cells with low expression of XRCC1 (a PARP1 partner in DNA repair) tended to be more sensitive to VE-821 but this was not statistically significant (*p* = 0.065), and it may be worth investigating in a larger panel of cell lines.

### 3.3. Increased Replication Stress Confers Sensitivity to Single Agent VE-821

Previous studies have shown that ATRi are more cytotoxic in cancer cell lines with increased RS [15,44,47,48,49]. Our data show that HGSOC cells were more sensitive to VE-821. HGSOC is characterized by genomic instability and DDR defects, which we hypothesized would increase the level of intrinsic RS. Therefore, we decided to assess the level of intrinsic RS across the panel of cell lines to establish if increased RS was associated with increased VE-821 sensitivity and hence responsible for the greater cytotoxicity in the HGSOC cells.

**Figure 3:**
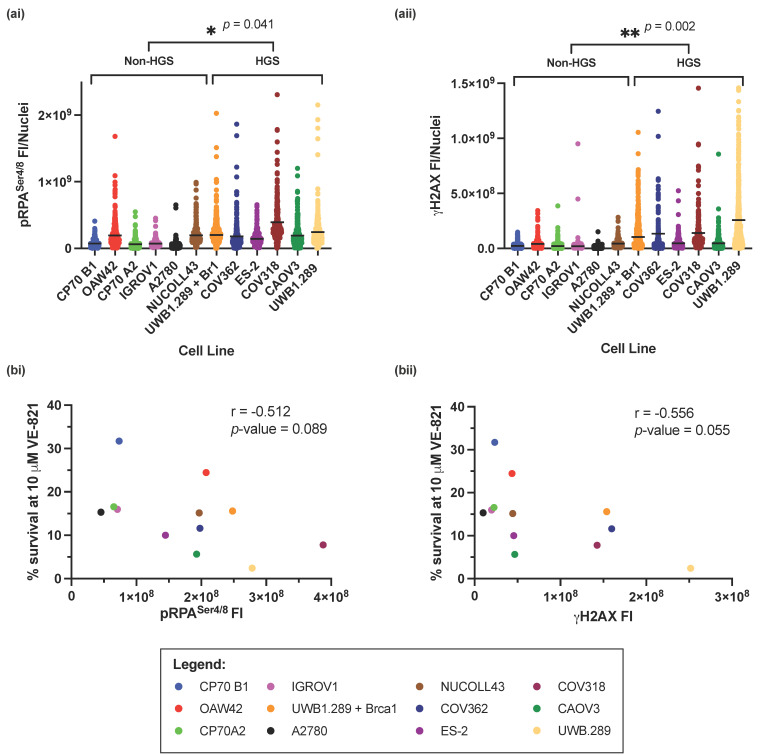
Measurement of intrinsic replication stress in the panel of ovarian cancer cell lines. (**a**) The fluorescence intensity of (**i**) pRPA^Ser4/8^ and (**ii**) γH2AX^Ser139^ from 3 independent experiments. Horizontal black line indicates the mean FI of either (**i**) pRPA or (**ii**) γH2AX^Ser139^ in each cell line. At least 80 nuclei were analyzed in each cell line in each experiment. Statistical significance between HGS and non-HGS cell lines * *p* < 0.05; ** *p* < 0.01 (Mann–Whitney test) is indicated on the graph. (**b**) Pearson’s rank correlation between; mean percentage cell survival at 10 μM VE-821 and mean (**i**) pRPA^Ser4/8^ and (**ii**) γH2AX^Ser139^ FI.

The RS markers pRPA^Ser4/8^ and γH2AX were measured across the panel of cell lines by immunofluorescence (IF) microscopy (Supplementary Figure S3), and fluorescence intensity (FI) was calculated. HGSOC cell lines had significantly higher levels of RS, by measurement of pRPA^Ser4/8^ (*p*-value = 0.041) (Figure 3(ai)) or γH2AX (*p*-value = 0.002) (Figure 3(aii)) suggesting that RS intrinsic to cell lines representative of HGSOC results in increased sensitivity to VE-821. However, overall, there was a negative correlation between cell survival at 10 µM VE-821 and pRPA^Ser4/8^ (Pearson’s correlation coefficient; r = −0.512, *p*-value = 0.089) (Figure 3(bi)), and γH2AX (Pearson’s correlation coefficient; r = −0.565, *p*-value = 0.055) (Figure 3(bii)) suggesting that independent of cell subtype, increased RS is a predictor of sensitivity to VE-821. Furthermore, 1 μM VE-821 potentiates the cytotoxicity of cells to cisplatin after induction of RS in IGROV1 and CAOV3 cells (Supplementary Figure S4).

pRPA^Ser4/8^ and γH2AX measure slightly different aspects of RS. Whilst pRPA^Ser4/8^ measures the accumulation of ssDNA, an early event in RS onset, γH2AX measures the development of DSBs, a probable consequence of persistent RS. Despite this, levels of pRPA^Ser4/8^ and γH2AX correlated well with each other across the panel of cell lines (Supplementary Figure S2, Pearson’s correlation coefficient; r = −0.744, *p*-value = 0.004).

## 4. Discussion

Multiple ATRi are currently in clinical trial as monotherapy and in combination with chemotherapeutics, radiotherapy, PARPi or immune checkpoint inhibitors. The development of these molecularly targeted drugs has driven the search for predictive biomarkers for patient treatment stratification. Whilst preclinical data are essential to identify and develop potential predictive biomarkers, much of it is based on studies using genetic modification of a single gene, which does not reflect the clinical situation where multiple determinants of resistance and sensitivity may be expressed in the same tumor.

We discovered that HGSOC cell lines were more sensitive to single agent VE-821 and focused our efforts on establishing why. Almost all HGSOCs are *TP53* mutant. p53 has been described as a determinant of sensitivity to ATR inhibition, although data has been conflicting with some studies reporting that p53 dysfunction is a determinant of sensitivity to ATRi [15,16,17] whilst others report that it is not [18,50]. Despite HGSOC cells being more sensitive to VE-821, in the cell lines studied here *TP53* mutation did not correlate with sensitivity to VE-821. Therefore p53 status may be a better determinant of chemo- or radio-sensitization to ATR inhibition, than ATRi monotherapy [18].

The most powerful determinant of sensitivity to ATR inhibition described pre-clinically and clinically is ATM dysfunction [17,19,20,21,22,42,51]. Recent results from phase I clinical studies have demonstrated durable anti-tumor activity of ATR inhibitors in advanced cancers with either deleterious *ATM* mutations or loss of ATM protein expression [52,53]. However, it should be noted that whilst *ATM* mutations are common in hematological malignancies, it has been predicted only 1–5% ovarian cancers contain somatic *ATM* mutations [5,54]. In this panel of cell lines, only the IGROV1 cells previously characterized as a hypermutated cell line [38] contained a heterozygous mutation in *ATM*; therefore, *ATM* mutation was not investigated as a determinant of sensitivity to VE-821. Despite this, ATM protein expression varied considerably across the panel of cell lines with an 18-fold difference in expression between the cells with the lowest ATM expression (CAOV3) and the highest expression (A2780). However, there was not a significant association between ATM expression levels and sensitivity to VE-821 in the panel of cell lines. This is in keeping with a recent study which found no association between *ATM* mutations and sensitivity to ATRi AZD6738, whilst ATM function did predict sensitivity to the drug [55]. Furthermore, a recently published phase II study of gemcitabine alone or gemcitabine + berzosertib (M6620) which retrospectively assessed the level of ATM expression by IHC, revealed that the addition of berzosertib to gemcitabine was beneficial irrespective of ATM protein expression [56,57]. Therefore, the value of ATM as a predictive biomarker is more complex than the literature once suggested.

RAD51 inhibition by the small molecular inhibitor BO2 was previously shown to significantly sensitize cells to the ATR inhibitor VE-821 [28], and HRR defects have also been identified as determinants of sensitivity to ATR inhibition in other studies [25,47]. Low RAD51 protein expression was identified as a determinant of sensitivity to VE-821 in this panel of cell lines. However, homozygous loss of *BRCA1* was also found to confer sensitivity to VE-821 but only in the context of an isogenic cell line pair, suggesting that whilst HRR defects may be predictive of sensitivity to ATRi, *BRCA* genetic testing alone may not be sufficient for patient stratification.

Additional proteins identified as potential determinants of sensitivity to VE-821 in this panel of cell lines were APOBEC3B and TopBP1. APOBEC3B, belonging to a subclass of cytidine deaminases responsible for converting cytosine to uracil during RNA editing and retrovirus or retrotransposon restriction, have previously been linked to increased levels of RS and mutagenesis [58], and overexpression of both APOBEC3A and 3B was found to increase RS and selectively sensitize to ATR inhibition [32,33]. However, in this panel of cell lines the reverse was seen; cells with low APOBEC3B tended to be more sensitive to VE-821 and have decreased levels of RS as measured by pRPA^Ser4/8^ and γH2AX (although not statistically significant). Preclinical data suggesting APOBEC as a determinant of sensitivity to ATR inhibition has led to investigation of APOBEC in clinical trials with a subset of patients with mutations in *APOBEC* genes being investigated in the OLAPCO clinical trial (NCT02576444) investigating olaparib in combination with ATRi AZD6738, the results of which may further elucidate the significance of APOBEC as a predictive biomarker.

Previous studies reporting RS as a determinant of sensitivity to ATRi have assessed oncogene-induced RS by MYC [48,49], or overexpression of cyclin E [15,59,60,61]. Here, we investigated the relationship between cyclin E protein expression (expected to be amplified in ~20% HGSOCs [5]) and sensitivity to VE-821. However, whilst the relationship between the two was not significant, there was an unexpected trend towards cells with decreased cyclin E being more sensitive to VE-821. We hypothesized that cells with increased cyclin E may have adapted to an increased level of RS and therefore may be better equipped to deal with the consequences of ATR inhibition and survive, however there was no statistically significant relationship between cyclin E expression and RS measured by pRPA^Ser4/8^ and γH2AX.

Given the difficulty in translating single previously identified determinants of sensitivity to VE-821, we decided to assess the level of intrinsic RS by IF microscopy. Previous functional assessment of replication fork stability in short-term patient derived HGSOC organoids, indicated that replication fork instability is associated with increased sensitivity to berzosertib [47]. Here, we showed that independently of ovarian cancer cell phenotype, cell lines with increased levels of RS (pRPA^Ser4/8^ or γH2AX) were significantly more sensitive to VE-821. Cells lines representative of HGSOC were found to have significantly higher levels of RS, which could underlie their increased sensitivity to VE-821. Whilst pRPA^Ser4/8^ and γH2AX independently predicted sensitivity to VE-821, there was a more significant association between γH2AX FI and sensitivity to VE-821. γH2AX is the primary response to DSBs and is phosphorylated by ATR in S-phase in response to RS and collapsed replication forks [62], as well as by ATM and DNA-PK in response to DSBs in other phases of the cell cycle [63], therefore γH2AX may be a reliable biomarker of ATRi response, coupled with ATR dependence. IHC has previously been employed to successfully measure γH2AX and pRPA^Ser33^ in triple negative breast cancer [61], and γH2AX in ovarian cancer [64]. Therefore, to translate this predictive biomarker to the clinic, IHC of pRPA^Ser4/8^ and γH2AX as well as high-throughput quantitative imaging analysis should be assessed and validated.

Whilst assessment of a single predictive biomarker, e.g., *TP53*, *ATM*, *BRCA1* may be more easily translated to the clinic, this study demonstrates that this approach may not be reliable when faced with complex cancer phenotypes. In conclusion, we propose RS may be a better predictive biomarker of ATRi monotherapy response than any single gene or protein in ovarian cancers.

## Supplementary Materials

The following supporting information can be downloaded at: https://www.mdpi.com/article/10.3390/cells11152361/s1. Table S1: LC_50_ of VE-821 and survival at 10 μM VE-821; Table S2: Comparison of LC_50_ of VE-821 and berzosertib; Figure S1: (a) Median VE-821 LC_50_ of HGS and non-HGS cell line is indicated with horizontal black line. ** *p* < 0.01 (Mann–Whitney test). (b) Median VE-821 LC_50_ of *TP53* WT or mutant cell lines is indicated by horizontal black line. ns, *p* > 0.05 (Mann–Whitney test).; Figure S2: Quantified expression of previously identified determinants of sensitivity to VE-821; Figure S3: Representative images of DAPI, pRPA^Ser4/8^ and γH2AX^Ser139^ IF staining in exponentially growing untreated IGROV1; Figure S4: Cisplatin cytotoxicity is potentiated by 1 μM VE-821 after induction of replication stress. (a) Western blot of IGROV1 and CAOV3 cells treated with 3 μM Cisplatin or 1 μM VE-821 for 24 h. (b) Potentiation of cisplatin cytotoxicity with 1 μM VE-821. Figure S5: Pearson’s correlation between mean pRPA^Ser4/8^ and γH2AX fluorescence intensity.
