# Supplementary material for: The Role of ATR Inhibitors in Ovarian Cancer: Investigating Predictive Biomarkers of Response
Source: Cells. 2022 Aug 1;11(15):2361. doi: 10.3390/cells11152361 (PMC9367423; doi:10.3390/cells11152361)
Supplement: Supplementary file 1 [file cells-11-02361-s001.zip › cells-1815551-supplementary.pdf]

Supplementary Table S1: LC_{50} of VE-821 and cell survival at 10 μM VE-821.

Cell Line	LC_{50} (μM) ± SEM	Cell survival at 10 μM VE-821 (% control) ± SEM
CP70 B1	4.91 ± 0.48	31.72 ± 1.92
NUCOLL43	2.12 ± 0.34	15.14 ± 2.58
IGROV1	1.90 ± 0.39	15.99 ± 4.76
CP70 A2	1.83 ± 0.24	16.56 ± 5.23
OAW42	1.53 ± 0.56	24.44 ± 7.64
COV362	1.50 ± 0.17	11.60 ± 2.26
A2780	1.34 ± 0.28	15.32 ± 2.35
COV318	1.14 ± 0.24	7.78 ± 2.93
ES-2	1.06 ± 0.13	10.02 ± 3.68
UWB1.289 + Brca1	0.96 ± 0.10	15.58 ± 1.41
CAOV3	0.78 ± 0.06	5.65 ± 1.46
UWB1.289	0.64 ± 0.06	2.43 ± 0.21

Supplementary Table S2: Comparison of LC_{50} of VE-821 and Berzosertib.

Cell Line	VE-821 LC_{50} (μM) ± SEM	Berzosertib LC_{50} (nM) ± SEM	Fold difference
IGROV1	1.90 ± 0.39	64.09 ± 7.37	30
CAOV3	0.78 ± 0.06	18.21 ± 2.60	43

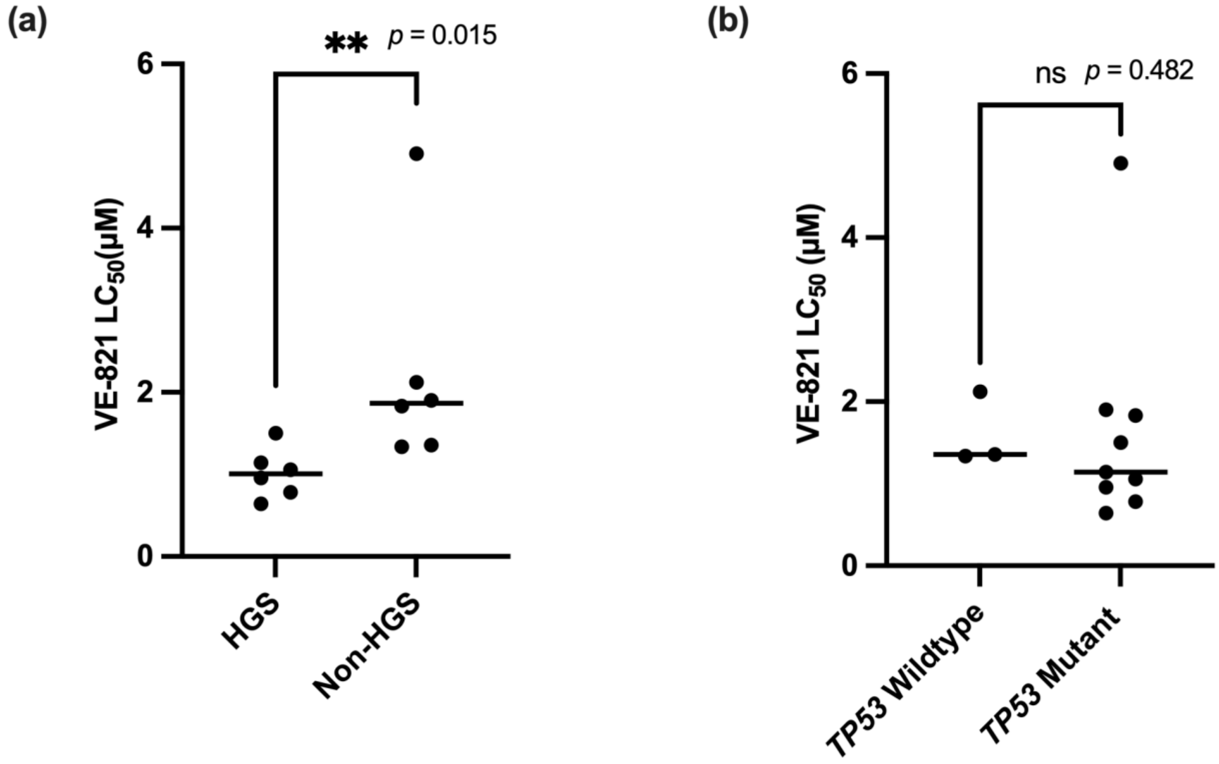

Supplementary Figure S1: (**a**) Median VE-821 LC_{50} of HGS and non-HGS cell line is indicated with horizontal black line. **, *P* < 0.01 (Mann-Whitney test). (**b**) Median VE-821 LC_{50} of *TP53* WT or mutant cell lines is indicated by horizontal black line. ns, *P* > 0.05 (Mann-Whitney test).

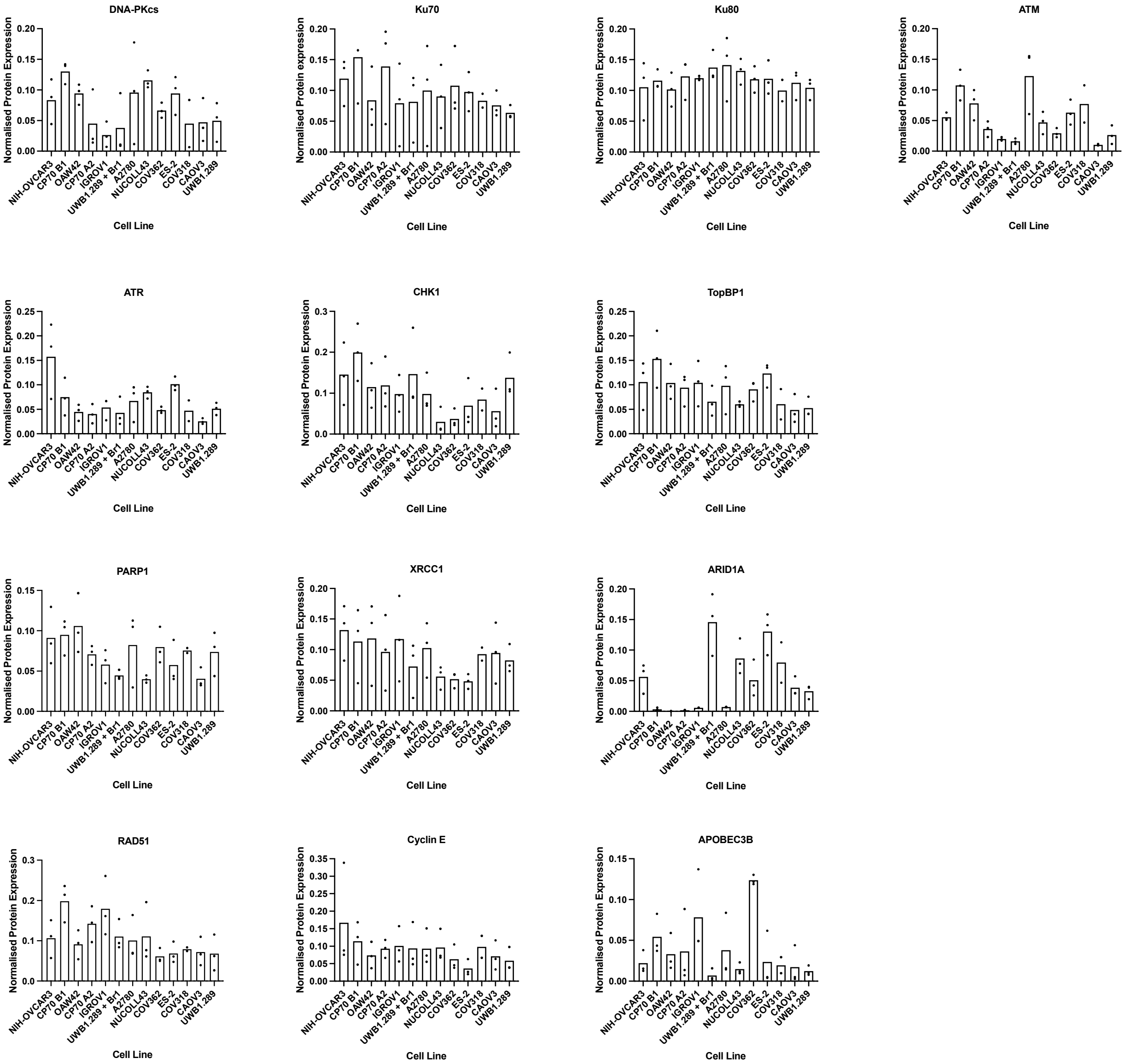

Supplementary Figure S2: Quantified expression of previously identified determinants of sensitivity to VE-821.

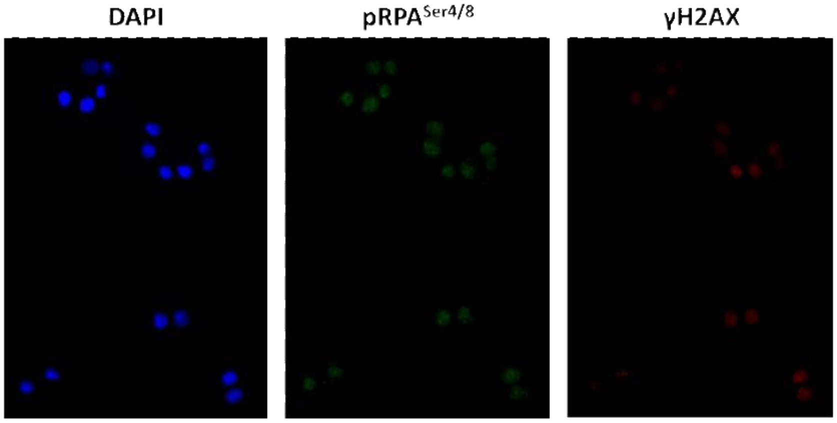

Supplementary Figure S3: Representative images of DAPI, $pRPA^{Ser4/8}$ and $\gamma H2AX^{Ser139}$ IF staining in exponentially growing untreated IGROV1.

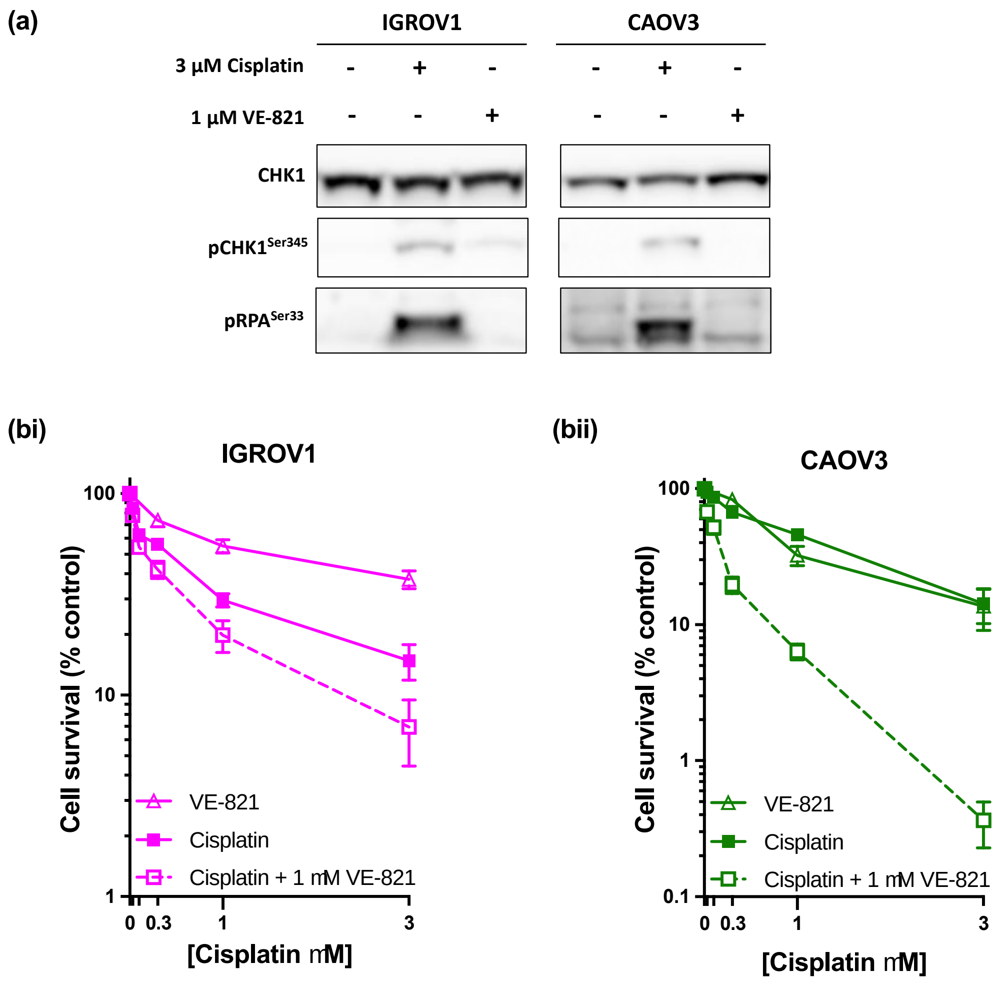

Supplementary Figure S4: Cisplatin cytotoxicity is potentiated by 1 µM VE-821 after induction of replication stress. (**a**) Western blot of IGROV1 and CAOV3 cells treated with 3 µM Cisplatin or 1 µM VE-821 for 24 hours. (**b**) Potentiation of cisplatin cytotoxicity with 1 µM VE-821.

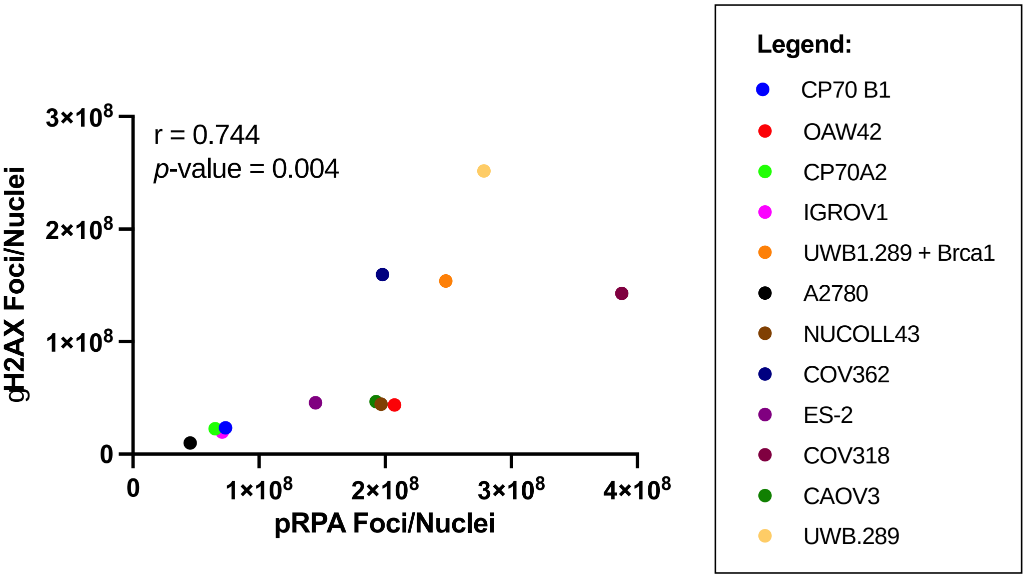

Supplementary Figure S5: Pearson's correlation between mean $pRPA^{Ser4/8}$ and $\gamma H2AX^{Ser139}$ fluorescence intensity.
